# Joint modeling of success and treatment discontinuation in *in vitro* fertilization programs: a retrospective cohort study

**DOI:** 10.1186/1471-2393-12-77

**Published:** 2012-08-03

**Authors:** Pénélope Troude, Sophie Ancelet, Juliette Guibert, Jean-Luc Pouly, Jean Bouyer, Elise de La Rochebrochard

**Affiliations:** 1 Ined, 133 boulevard Davout, F-75020, Paris, France; 2 Inserm, CESP U1018, Site de Bicêtre, 82 rue du Général Leclerc, F-94276, Le Kremlin-Bicêtre, France; 3 Univ Paris-Sud, UMRS 1018, F-94276, Le Kremlin-Bicêtre, France; 4 Institute for Radiological Protection & Nuclear Safety, Laboratory of Epidemiology, BP 17, 92262, Fontenay-aux-Roses Cedex, France; 5 Unité de Médecine de la Reproduction, Service de Gynécologie-Obstétrique II, Hôpital Cochin, 27 rue du Faubourg Saint-Jacques, F-75014, Paris, France; 6 Laboratoire de Procréation Médicalement Assistée, Institut Mutualiste de Montsouris, 42 boulevard Jourdan, F-75014, Paris, France; 7 Unité de FIV, CHU de Clermont-Ferrand, 58 rue Montalembert, F-63003, Clermont-Ferrand, France

## Abstract

**Background:**

As discontinuation in *in vitro* fertilization (IVF) programs has been associated with a poor prognosis, one hypothesis is that some couple-specific predictive factors in IVF may be shared with opposite effect by both success (i.e. live birth) and treatment discontinuation processes. Our objective was to perform a joint analysis of these two processes to examine the hypothesis of a link between the two processes.

**Methods:**

Analyses were conducted on a retrospective cohort of 3,002 women who began IVF between 1998 and 2002 in two French IVF centers: a Parisian center and a center in a medium-sized city in central France. A shared random effects model based on a joint modelization of IVF treatment success and discontinuation was used to study the link between the two processes.

**Results:**

Success and discontinuation processes were significantly linked in the medium-sized city center, whereas they were not linked in the Parisian center. The center influenced risk of treatment discontinuation but not chance of success. The well-known inverse-J relation between the woman’s age and chance of success was observed, as expected. Risk of discontinuation globally increased as the woman’s age increased.

**Conclusions:**

The link between success and discontinuation processes could depend on the fertility center. In particular, the woman’s decision to pursue or to discontinue IVF in a particular center could depend on the presence of other IVF centers in the surrounding area.

## Background

Discontinuation of *in vitro* fertilization (IVF) treatment is common, whatever the technical or social management of patients [1-3]. During recent years, studies conducted in various countries such as the Netherlands, Sweden and the United Kingdom have reported very high rates of discontinuation in IVF programs, with 25% to 50% of couples discontinuing treatment after the first or second attempt [1,2,4-6]. In countries where the financial costs of IVF must be mainly borne by the couple, such as the United Kingdom and the United States, the decision to continue or to discontinue IVF treatment is probably strongly influenced by financial issues [3]. In France, where couples receive financial support for IVF treatment (limited to 4 attempts for one pregnancy), discontinuation rates also appear to be high, with estimated cumulative discontinuation rates of 46% to 58% before the fourth attempt [7,8]. Treatment discontinuation is thus not only a financial matter and may be motivated by the heavy psychological or physical burden of IVF treatment and/or by a poor prognosis [2,3].

In an English cohort study of 2,056 couples, the characteristics of patients who discontinued IVF treatment after the first attempt were compared with those who had a second IVF attempt [1]. Among couples who discontinued IVF treatment, more women were aged >35 years, had five or less oocytes retrieved at the first attempt and two or less embryos available at the first attempt [1]. All these factors were associated with a lower chance of successful IVF. Other studies also demonstrated common factors associated with success and treatment interruption [4,6,9,10]. Some predictive factors in IVF treatment thus appeared to be shared by both success and treatment discontinuation processes, having an opposite impact on the two processes. For example, as the woman’s age increases, the chance of success decreases and, at the same time, the risk of treatment interruption increases. Such a link between success and treatment interruption is confirmed by studies investigating reasons for IVF treatment discontinuation. Even if the reason for treatment interruption is difficult to assess (it is probably a multifactorial decision, and one that is often studied several years after interruption), these studies showed that one in four couples considered that their treatment interruption was due to a poor prognosis [2,3]. Such results demonstrate the importance of studying discontinuation and success processes together.

Our aim was to investigate conjointly treatment discontinuation and success processes in two IVF centers and to examine the hypothesis that there is a link between the two processes in each center. We also aimed to explore the effects of the woman’s age and of the IVF center on the success process and on the discontinuation process. For this purpose, a shared random effects model was used.

## Methods

### Design and subjects

The study was conducted in two French IVF centers: a center located in Paris (Cochin) and a center located in a medium-sized city in central France (Clermont-Ferrand).

All women having their first aspiration in one of the two centers between 1998 and 2002 were included in the study (*n* = 3,037). Thirty-five women were excluded because the result of the first attempt was unknown, leaving 3,002 women. This study received approval from the French Data Protection Authority in September 2005 (authorization number 05-1334).

Information was collected from medical records for all aspirations undergone by the couples in the IVF center, as well as data on frozen embryo transfer (FET), up to 2005. The couples’ characteristics collected included the woman’s date of birth, date of aspiration, number of oocytes retrieved, IVF technique used, number of fresh embryos transferred, number of frozen embryos and the result of transfer (pregnancy, delivery). As the French social security system reimburses IVF treatment up to four aspirations, data collection was discontinued after the woman’s fourth aspiration.

### Outcome measures

The success of IVF was measured by a live birth after one attempt. The live birth rate was defined as a delivery resulting from fresh or frozen embryo transfer among women who had undergone one IVF attempt. Treatment discontinuation was defined as no treatment for at least two years in the IVF center (whatever the reason for discontinuation, e.g. maternal age, financial resources, move to another area, seeking IVF treatment elsewhere…) [8,11,12]. Discontinuation rate was defined as discontinuation among women who had not obtained a live birth after the IVF attempt.

### Descriptive statistics

Women’s characteristics at the first attempt were compared according to inclusion center, using the chi 2 test. Live birth rate and discontinuation rate at each attempt were also compared according to inclusion center.

### Shared random effects model

Success (live birth) and treatment discontinuation shared factors that underlie a couple’s susceptibility to both events (with opposite impact). These underlying shared factors may represent for example psychological factors and are difficult to measure. Conventional models (such as Cox proportional hazards models or multinomial models) do not make it possible to include such unmeasured shared factors. The concept of the shared random effects model is to include a random effect representing these shared unmeasured factors that impact on the two processes. The shared random effects approach has been described as “a very intuitive appeal to biomedical researchers who generally believe that there may be some latent quantity underlying a person’s susceptibility to both disease and death” [13]. By analogy, in our study “disease” is IVF success, and “death” is treatment discontinuation. Thus, we used a shared random effects model [14], composed of two mixed logistic regression models, one for success (*p*_*i*_) and one for discontinuation ( $\pi_{i}$) where *i* represented the couple:

(1) $$\log it(p_{i})=\alpha^{\mathrm{succ}}+\beta_{age_{i}}^{\mathrm{succ}}+\beta_{center_{i}}^{\mathrm{succ}}+f_{i}$$

(2) $$\log it(\pi_{i})=\alpha^{\mathrm{disc}}+\beta_{age_{i}}^{\mathrm{disc}}+\beta_{center_{i}}^{\mathrm{disc}}+\lambda f_{i}+{ɛ}_{i}$$

These models included baseline factors ( $\alpha^{\mathrm{succ}}$ and $\alpha^{\mathrm{disc}}$). They included two observed determinants called “fixed effects”:

the IVF center (Parisian center/medium-sized city center) denoted $\beta_{center_{i}}^{\mathrm{succ}}$ and $\beta_{center_{i}}^{\mathrm{disc}}$

and the woman’s age at the first attempt divided into five classes (age <25/25-29/30-34/35-39/≥40 years) denoted $\beta_{age_{i}}^{\mathrm{succ}}$and $\beta_{age_{i}}^{\mathrm{disc}}$.

It also included a “random effect” (*f*_*i*_) that is common to both mixed logistic regression models. This shared “random effect” represents all the couple-specific and non-explicitly identified factors explaining both processes. The coefficient associated with this shared random effect (λ), could be interpreted as the “link” between the two processes: if λ equals zero, it means that there are no couple-specific factors (other than the observed covariates included in the model) that simultaneously explain IVF success and treatment discontinuation. On the contrary, if λ differs from zero, the interpretation is that there are couple-specific non-observed factors that determine both IVF success and treatment discontinuation. When λ is strictly negative, it means that these factors have an opposite relative impact on the two processes. In our model, a specific λ was considered for each center (λ_Paris_ and λ_Medium-sized city_) in order to allow different degrees of “links” between the two processes in the two IVF centers. Detailed information on this model is given in the Additional file 1.

The shared random effects model was fitted with Bayesian computational methods using Markov Chain Monte Carlo (MCMC) [15] and implemented in WinBUGS [16]. We ran two independent MCMC chains (using different initial values for the parameters) of 300,000 simulations with a burn-in period of 50,000 and kept every 100^th^ to reduce autocorrelation in the MCMC samples. Our results are therefore based on thinned samples of size 5,000. Convergence of the MCMC run was assessed by graphical inspection of the chains and by computing the Gelman-Rubin statistics as modified by Brooks and Gelman [17,18] and intra-chains autocorrelations. Credible intervals (95%) were estimated. The λ_Paris_ and λ_Medium-sized city_ parameters were tested according to Bayesian statistical theory methods using partial Bayes factors (BF) [19]: no evidence that λ ≠ 0 if L = 2logBF is greater than -2, λ significantly different from 0 if L ≤ -2 and strongly different from 0 if L ≤ -6.

We performed a Bayes factor sensitivity analysis to prior choice of parameter distribution [19] and checked the stability of our Bayesian method [20]. We computed partial Bayes factors from more or less informative priors (and only one MCMC run) by performing a split test sample analysis as described elsewhere [20]. We considered three ways to split the sample of 3,002 women so as to define a learning sample (i.e., 2,702, 1,502 and 502 women) that would be more or less informative on the remaining test sample (i.e., 300, 1,500, 2,500 women).

## Results

The characteristics of the study population at the first aspiration are described in Table 1. The proportions of patients in each of the two centers were similar, with 1,556 women having a first aspiration in the Parisian center between 1998 and 2002 and 1,446 in the medium-sized city center. Globally, median age at first aspiration was 32 years. The women treated in the Parisian center were somewhat older than women treated in the medium-sized city center (median age 33 vs 32 years), had more frozen embryos (p < 0.001) and slightly fewer fresh embryos transferred (p < 0.001). The ICSI technique was used more frequently in the Parisian center than in the medium-sized city center (p < 0.001).

**Table 1:** **Characteristics of study population at the first aspiration (*N *** **= 3,002)**

	**Parisian center (Cochin)**	**Medium-sized city center (Clermont-Ferrand)**	P*
	***n *=1,556 (%)**	***n *=1,446 (%)**	
**Patient age (years)**			<0.001
17-24	2	3	
25-29	21	27	
30-34	39	41	
35-39	29	23	
≥ 40	9	6	
**Technique**			<0.001
IVF	48	61	
ICSI	52	39	
**Oocytes retrieved**			0.054
0	1	2	
1-6	34	35	
7-15	50	47	
16-60	15	16	
**Fresh embryos transferred**			<0.001
0	13	13	
1	10	13	
2	65	52	
3-5	12	22	
**Embryos frozen**			<0.001
0	49	70	
1-2	21	13	
3-21	30	18	

* p-value for the chi2 test comparing the women’s characteristics according to inclusion center.

Global observed cumulative success rate (live birth) was 37% (1,107/3,002); for the Parisian center, it was 34% and for the medium-sized city center 41% (p < 0.001). Live birth rates per attempt decreased with increasing number of attempts (Table 2) from 22% for the first attempt in the Parisian center to 9% for the fourth, and from 21% to 16% for the medium-sized city center. Globally, 48% of women discontinued IVF treatment (54% in the Parisian center, 42% in the medium-sized city center, p < 0.001). The proportion of treatment discontinuation increased with the increasing number of attempts and was significantly higher in the Parisian center at each attempt.

**Table 2:** **Observed live birth and discontinuation rates in the study population (*N*** **= 3,002)**

	**Parisian center (Cochin)**	**Medium-sized city center (Clermont-Ferrand)**	P*
	***n *=1,556 (%)**	***n *=1,446 (%)**	
**Live birth rate**			
1^st^ attempt	22	21	ns
2^nd^ attempt	18	19	ns
3^rd^ attempt	9	17	0.002
4^th^ attempt	9	16	0.066
**Discontinuation rate**			
1^st^ attempt	37	25	<0.001
2^nd^ attempt	46	31	<0.001
3^rd^ attempt	49	39	0.010

* p-value for the chi2 test comparing live birth rate and discontinuation rates according to inclusion center.

The statistical “link” between the success and the treatment discontinuation processes was estimated separately for each center with two coefficients being introduced in the model, one for each center: λ_Paris_ and λ_Medium-sized city_. Estimated λ_Paris_ was very close to 0 whereas that of λ_Medium-sized city_ was -0.21 (Table 3). The 95% credible interval for λ_Medium-sized city_ was skewed toward negative values ([-0.5,0.0]) whereas that of λ_Paris_ was centered on 0 ([-0.2,0.2]). Comparison of models (Table 3, see notes) showed that there was no evidence of a link between the success and discontinuation processes in the Parisian center, whereas there was a significant negative link in the medium-sized city center. Finally, Bayes factor sensitivity to prior choices showed the stability of these results. Indeed, λ_Paris_ always appeared as non-significantly different from 0 and λ_Medium-sized city_ always appeared as significantly (and sometimes even strongly significantly) different from 0.

**Table 3:** Estimations of the links between the success and treatment discontinuation processes for the Parisian and the Medium-sized city center

	**Posterior mean (posterior standard deviation)**^** a**^	**95% credible interval**	**Test of λ**^** b**^
**λ**_**Paris**_	0.01 (0.11)	[-0.2,0.2]	L = 2.70 ^c^ - *NS*
**λ**_**Medium-sized city**_	-0.21 (0.12)	[-0.5,0.0]	L = -5.26 ^d^ - *S*

^a^ the models were adjusted for female age and center.

^b^ using partial Bayes factors (see Methods section).

^c^ The competitive models were M_0_: *[λ*_*Paris*_ *= 0 and λ*_*Medium-sized city*_*=0]* vs M_1_: *[λ*_*Paris*_ *≠ 0 and λ*_*Medium-sized city*_*=0 ].* There is a positive evidence against λ_Paris_ ≠ 0 meaning that λ_Paris_ is non-significantly (NS) different from 0 (H_0_ is not rejected).

^d^ The competitive models were M_0_: *[λ*_*Paris*_ *= 0 and λ*_*Medium-sized city*_ *= 0]* vs M_1_: *[λ*_*Paris*_ *= 0 and λ*_*Medium-sized city*_ *≠ 0*]. There is a positive evidence against λ_Medium-sized city_ =0 meaning that λ_Medium-sized city_ is significantly (S) different from 0 (H_0_ is rejected).

**Figure 1:**
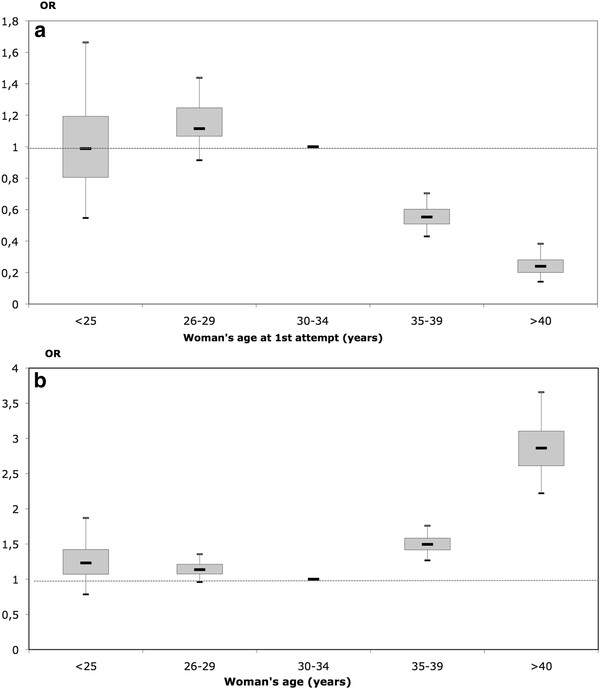
**Estimated OR and boxplot **^**a **^**of the effect of the woman’s age on the success and treatment discontinuation processes (reference age group was 30-34 years).a.** Success **b.** Treatment discontinuation ^a^ median, upper and lower quartiles, and 95% credible interval. Note: *Bayesian estimations provide estimation of the distribution of the odds ratio (OR) and not one single punctual estimate. Consequently, a box-plot is used to describe the estimated distribution of the OR with its quantiles 0.025, 0.25, 0.5 0.75 and 0.975.*

Using this model, we studied the relationship between success, discontinuation and the woman’s age. Results are presented in Figure 1, with the 30-34 year old group as reference. The chance of success varied as an inverse J-shape with a maximum at age 25-29 years and a strong decrease among older women (Figure 1a). The risk of treatment discontinuation varied in the opposite direction with a minimum at age 30-34 years and a strong increase among older women (Figure 1b).

**Figure 2:**
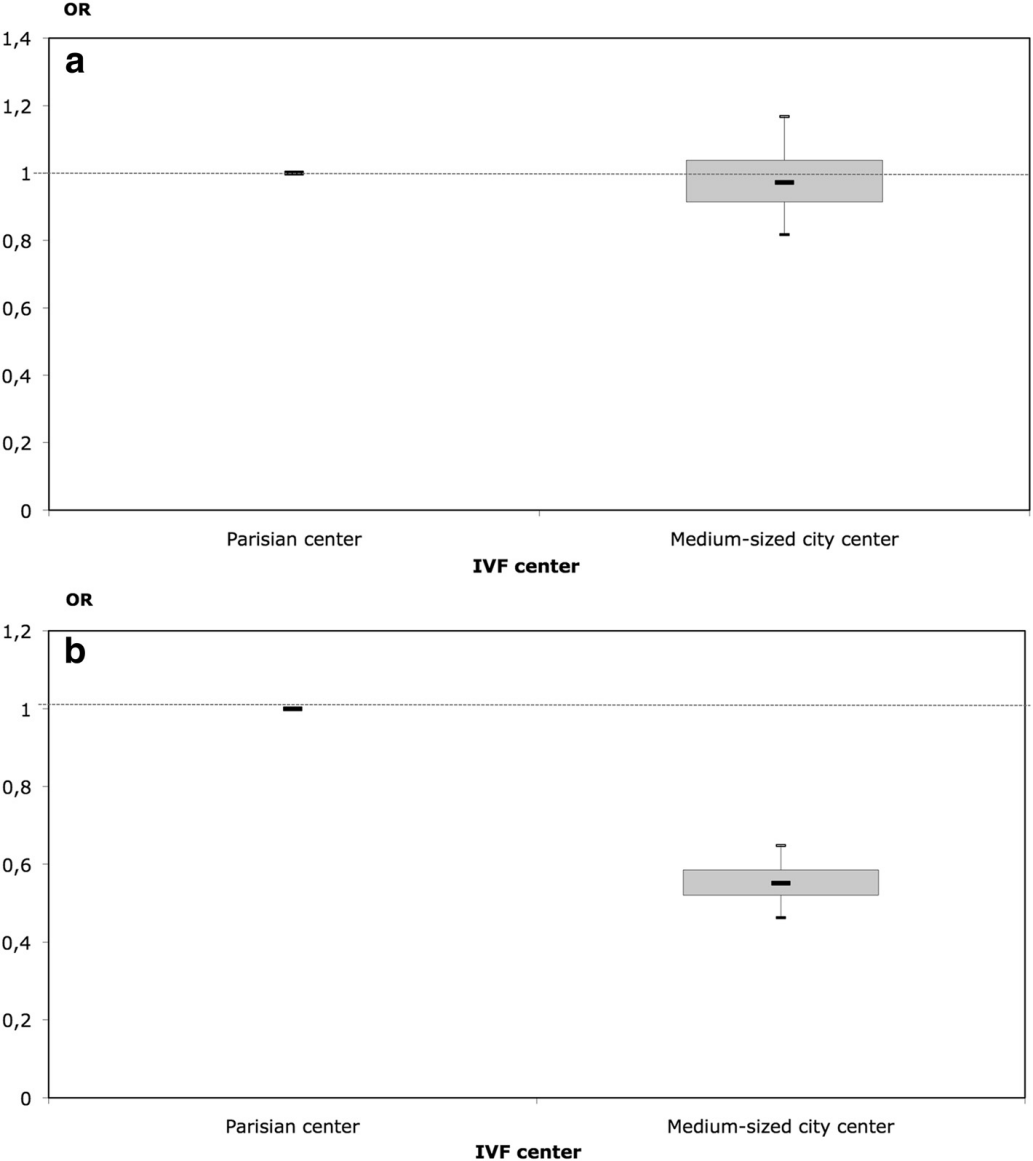
**Estimated OR and boxplot **^**a **^**of the effect of the IVF center on the success and treatment discontinuation processes (Parisian center as reference).a.** Success **b.** Treatment discontinuation. ^a^ median, upper and lower quartiles, and 95% credible interval. Note: *Bayesian estimations provide estimation of the distribution of the odds-ratio (OR) and not one single punctual estimate. Consequently, a box-plot is used to describe the estimated distribution of the OR with its quantiles 0.025, 0.25, 0.5 0.75 and 0.975.*

The associations between IVF center and success and treatment discontinuation are presented in Figure 2, with the Parisian center as reference. The probability of success did not differ according to IVF center (OR of success was 0.97 with 95% credible interval [0.8;1.2]), whereas women treated in the medium-sized city center had a lower risk of treatment discontinuation than women treated in the Parisian center (OR of treatment discontinuation was 0.55 with 95% credible interval [0.5;0.7]).

## Discussion

To study jointly success and treatment discontinuation in IVF programs, a shared random effects model was built and used to analyze data from two French IVF centers: a Parisian center and a medium-sized city center. We found no evidence of a link between success and discontinuation processes in the Parisian center, whereas we did find one in the medium-sized city center. In the medium-sized city center, the negative link observed between the two processes meant that women who discontinued treatment in this center had a lower probability of success. The direction of the link was expected and is in agreement with the literature, as previous studies have reported poorer prognostic factors of IVF success among women who discontinued treatment [1,4,10]. However, it is interesting that no evidence of such a link was observed in the Parisian center.

### Woman’s age: effect on success and treatment discontinuation

By defining success as a live birth during the entire IVF program (first to fourth IVF attempts), we demonstrated an inverse-J relationship between the woman’s age and success. Such a relationship has already been shown [21]. We also found that the probability of discontinuation varied inversely to the probability of success according to the woman’s age, and that the probability of discontinuation globally increased with increasing female age. In our study, using women aged 30-34 years as reference, OR of treatment discontinuation was 2.9 ([2.2-3.7]) for women older than 40 years. A few studies have already shown that women who discontinued treatment were generally older that women who persevered [22]. In our study, we assessed how the cumulative risk of discontinuation alters with the woman’s age and we demonstrated a J-relationship between the woman’s age and cumulative risk of discontinuation during an IVF program.

### IVF center: variability in success and treatment discontinuation

In our study, the crude cumulative success rate differed according to center (34% vs 41%), but after controlling for treatment discontinuation and the woman’s age, the probability of success no longer differed between centers (Figure 2). This result is similar to that of a study carried out in two centers in the Netherlands [23]. Investigators found that the crude cumulative live birth rates differed between the two centers. However, this difference was not due to differences in success rates at each attempt, but rather to different discontinuation rates in each center. These observations showed that comparison of IVF centers should not be done on crude success rate and that treatment discontinuation is an important factor that should be taken into account.

Conversely, we found that treatment discontinuation rates differed between centers, being lower in the medium-sized city center. Moreover, a negative link was found between success and discontinuation in the medium-sized city center, whereas there was no evidence of such a link in the Parisian center. One explanation could be that the characteristics of women who discontinued treatment differed between the two centers. In the medium-sized city center, the negative link meant that women who discontinued had poorer prognostic factors. In the Parisian center, there was no reason why women with poorer prognostic factors did not discontinue treatment, but the higher level of discontinuation could indicate that women with good prognostic factors also discontinued IVF treatment in the Parisian center. This hypothesis of a more mixed population could explain the lack of significant link between success and discontinuation in the Parisian center. Differences between fertility centers may be linked to various factors such as patient selection, medical staff, or management practices (i.e. choice of IVF vs ICSI, number of embryos transferred). However, one major difference between the two centers in our study is their geographical environment: the Parisian center is surrounded by 23 other IVF centers (9 in Paris itself and 14 in the suburbs), whereas the medium-sized city center is the only one in this administrative area and the nearest other center is in the city of Lyon, a 2-hour drive away. Consequently, the medium-sized city center could be defined as a monopoly center, whereas the Parisian one competes with several other fertility centers. When there are several fertility centers close to the woman’s place of residence (a competition situation between centers), the population of women who discontinue is probably mixed, consisting of both patients with a poorer prognosis and patients who merely change IVF center, whatever their prognosis. On the contrary, in a monopoly center, as women cannot easily discontinue in order to begin another IVF program elsewhere, most treatment discontinuations are linked to poorer prognostic factors.

To the best of our knowledge, this is the first time that the hypothesis of an association between treatment discontinuation and a monopoly/competition situation of the IVF center has emerged in the literature. However, an association between success and a monopoly situation or competition between IVF centers has already been considered. Indeed, some studies have tested the association between an increasing number of multiple pregnancies and competition between IVF centers, the underlying hypothesis being that a greater number of embryos are transferred in centers that compete against various others, in order to maximize the chance of success [24,25]. Recently, a large American study, conducted in clinics performing ART between 1995 and 2001 (*n* = 2374 clinic-years), has examined the relationship between competition and clinic-level ART outcomes and practice patterns [26]. Defining competition as the number of clinics within a 20-mile radius (32.19 km) of a given clinic, they found no evidence of a significant relationship between competition and birth rates in multivariate models. Moreover, they found a lower, rather than a higher, rate of multiple births per ART cycle for clinics in highly competitive areas, as has been suggested in one previous study using another definition for competition [27]. Our results are in agreement with the American study, showing no difference between the two centers, one being in a monopoly situation and the other in a competition situation, with regard to chance of success.

### Study limitations

In our model, we included only female age and center as fixed effects. In the context of growing interest in understanding differences between IVF success rates according to center [28,29], some studies have explored to what extent such differences may be linked to differences in patients’ characteristics. An English study has explored the influence of patients’ characteristics on live birth rate per cycle started [30]. The authors demonstrated the impact of non-IVF related patient characteristics on the success rate and concluded that using a “standard patient group and outcome” did not improve validity of comparisons between centers. More recently, using IVF and ICSI treatment data from 11 IVF centers in the Netherlands, Lintsen et al. studied how differences in IVF success rates between centers could be explained by patient characteristics and concluded that only 17% of the variation between centers could be explained by patient mix [31]. Thus, there is currently no clear evidence that other patient characteristics should be taken into account in our multivariate model. However, our shared random effects model could be extended by including temporal effects that could describe, for instance, the patient’s level of discouragement due to psychological and physical burden. Such a temporal effect could also be included in the model to test if the link between the success and treatment discontinuation processes may also depend on the IVF attempt. Obviously, it would be of great interest to conduct such analysis on a greater number of centers to better understand how the center’s situation impacts on treatment discontinuation.

### Study implications

Despite the increasing interest in understanding differences in IVF success rates between IVF centers, the reasons explaining such differences remain rather unclear. It is likely that differences in IVF centers success rates are a combination of patient and center characteristics [32]. Treatment discontinuation rate could be one of the factors impacting on the center success rate but it has scarcely been investigated. In our study, we observed two French centers with different crude success rates. After controlling for the woman’s age and for the impact of discontinuation on success rate in a shared random effects model, success rates between the two centers no longer differed. Our results showed that discontinuation may be a very important factor in explaining success rate differences between centers, and it needs to be better understood.

Our study also enabled us to explore treatment discontinuation. Our main result was that discontinuation appeared very dissimilar in the two centers. The center strongly influenced the risk of treatment discontinuation (unlike the chance of success). An important perspective of this work will be to explore further the discontinuation process and differences between centers in a larger number of centers. Based on our results, a very promising hypothesis would be to explore the possible influence of the IVF center situation (monopoly/non-monopoly) on the probability of treatment discontinuation. Our hypothesis is that the probability of treatment discontinuation decreased in centers that were in a monopoly situation.

## Conclusion

Based on IVF treatment, we hypothesize that a better understanding of the treatment discontinuation process in relation to competition may be very helpful in understanding differences in success rates between IVF centers. It would be very interesting to explore if a similar hypothesis would be pertinent for treatments other than IVF, when patients have the possibility of deciding to pursue their treatment in another medical center.

## Abbreviations

IVF: *In vitro* fertilization; OR: Odds ratio.

## Competing interest

None of the authors have a conflict of interest.

## Authors’ contributions

PT designed the study’s analytic strategy, conducted the literature review and drafted the manuscript. SA designed the study’s analytic strategy, conducted the statistical analysis, drafted the Methods section and reviewed the manuscript. JG coordinated the study in fertility centers, reviewed the study's analytic strategy and results, and reviewed the manuscript. JLP coordinated the study in fertility centers, reviewed the study's analytic strategy and results and reviewed the manuscript. JB designed the study’s analytic strategy, reviewed the results and reviewed the manuscript. EDLR designed the study, designed the study’s analytic strategy and drafted the manuscript. All authors read and approved the final manuscript.

## Pre-publication history

The pre-publication history for this paper can be accessed here:

http://www.biomedcentral.com/1471-2393/12/77/prepub

## Supplementary Material

Additional file 1 The appendix includes detailed information on the shared random effects model. Click here for file
