# Supplementary material for: Joint modeling of success and treatment discontinuation in in vitro fertilization programs: a retrospective cohort study
Source: BMC Pregnancy Childbirth. 2012 Aug 3;12:77. doi: 10.1186/1471-2393-12-77 (PMC3514282; doi:10.1186/1471-2393-12-77)
Supplement: Additional file 1 — The appendix includes detailed information on the shared random effects model. [file 1471-2393-12-77-S1.doc]

**APPENDIX**

This appendix provides details on our shared random effects model and the Bayesian methods implemented to fit the model.

**The shared random effects model**

The outcomes data are discrete survival data. The following binary outcomes have been defined for each couple i=1,…, 3,002 and IVF attempt j=1,2,3,4:

- IVF outcome (delivery or not) of the couple i at attempt j (only the first success being taken into account), denoted Y_ij_.

- Discontinuation between attempt j and j+1 for the couple i, denoted D_ij_. D_i1_ is defined only for the 2,352 couples who did not deliver at attempt 1.

The following conditional distribution was assumed for IVF outcome:

In the above expression,

- *p_i_* denotes the conditional probability that the i^th^ couple delivers at any attempt after j, given that this couple does not deliver prior to attempt j. This conditional probability is assumed to be the same for all the attempts undergone by couple i.
- $\alpha^{succ}$ is the overall baseline probability of delivery for the first time after any attempt.
- $\beta_{age_{i}}^{succ}$and $\beta_{center_{i}}^{succ}$are the vectors of success-specific “fixed” effect parameters that can be interpreted as log odds ratios corresponding to female age and the treatment center of couple i respectively.
- $f_{i}$is a couple-specific “random” effect that acts as a surrogate for some unmeasured IVF predictive factors of the couple i which, if they could be quantified, would have allowed better assessment of the probability of success for couple i.

The following initial conditions were Y_i0_=0 for all i (i=1,2,…, 3,002).

Similarly, the following conditional distribution was defined for the treatment discontinuation process:

$$D_{ij}\left|D_{ij'}=0j'<j\right.\sim^{i.i.d.} Bernoulli\left(\pi_{i}\right)$$

where:

- $\pi_{i}$ is the conditional probability that the i^th^ couple discontinues at any attempt j, given that this couple does not discontinue prior to attempt j. It is assumed to be the same for all the attempts undergone by couple i.
- $\alpha^{drop}$ is the overall baseline probability of treatment discontinuation after any attempt.
- $\beta_{age_{i}}^{drop}$and $\beta_{center_{i}}^{drop}$are the vectors of discontinuation-specific “fixed” effect parameters that can be interpreted as log odds ratios corresponding to female age and the treatment center of couple i respectively.
- λ is a scale parameter that allows weighting of the relative contribution of some unmeasured shared IVF predictive factors *f_i_* of the couple i to discontinuation risk compared with success risk. This plays a similar role to a regression coefficient and allows difference in the relative impact of the non-explicitly identified unmeasured IVF predictive factors shared by success risk and discontinuation risk.
- $\varepsilon_{i}$is a couple-specific “random” effect that acts as a surrogate for all the non-explicitly identified factors that only influence the risk of treatment discontinuation (e.g., financial burden).

The following initial conditions were D_i0_=0 for all i (i=1,2,…, 3,002).

In the above expressions, the *f_i_* are shared “random” effects as they simultaneously appear in the success and the treatment discontinuation process. Basically, they influence the couples’ susceptibility to both deliver and discontinue their treatment during an IVF program.

The following normal distributions were assigned to all the “random effects” to describe the between-couple variability, caused by some unmeasured IVF predictive factors, explaining either both the success and the discontinuation processes or only the discontinuation process:

$$f_{i}\sim^{i.i.d.} Normal(0,\sigma_{f}^{2})$$

$$\varepsilon_{i}\sim^{i.i.d.} Normal(0,\sigma_{\varepsilon}^{2})$$

where $\sigma_{f}^{2}$and $\sigma_{\varepsilon}^{2}$are variance parameters controlling such between-couple variability.

**Prior distributions**

As usual in the Bayesian framework, probability distributions, called “prior distributions”, must be assigned to all model parameters .

Normal priors with high variances (meaning that no precise prior knowledge is available) were assigned to the baseline risks $\alpha^{succ}$and $\alpha^{drop}$and all the “fixed” effect parameters . We assigned the rather vague uniform prior distributions Unif[0, 10] to $\sigma_{f}^{2}$and $\sigma_{\varepsilon}^{2}$ . Finally, the following uniform prior Unif[-10,10] was assigned to the λ_center_ scale parameters. This prior is flat as no precise prior knowledge is available for λ_center_ and it makes it possible to consider a plausible range of values for λ_center_.

**The Bayesian computational methods implemented**

The shared random effects model was fitted with Bayesian computational methods, namely Markov Chain Monte Carlo (MCMC) algorithms as implemented in WinBUGS . We ran two Markov chains (using different initial values for the parameters) of 300,000 simulations with a burn-in period of 50,000 and kept every 100^th^ to reduce autocorrelation in the MCMC samples. Our results are therefore based on thinned samples of size 5,000. Convergence of the MCMC run was assessed by graphical inspection of the chains and by computing the Brooks and Gelman statistic and also the ratio of the Monte Carlo (MC) error to the posterior standard deviation [13].

**Checking convergence of the MCMC algorithm**

Convergence of the MCMC algorithm is good for all parameters. Figure A displays the posterior distributions estimated for all the parameters from the two MCMC chains. We can see that they clearly overlap which emphasizes a good convergence. This is confirmed by Brooks and Gelman statistics that are somewhat above 1 but in all cases below 1.1.

Figure A also shows that the posterior distributions are far from being flat, meaning the prior beliefs have been updated by the data. Finally, the ratios of the Monte Carlo (MC) error to the posterior standard deviation are inferior to 0.05 for all the model parameters, which confirms that we have run the simulation for a sufficient number of iterations to obtain accurate posterior estimates.

**Figure A.** Comparison of the two posterior distributions obtained from two MCMC chains for the model parameters and some random effects.


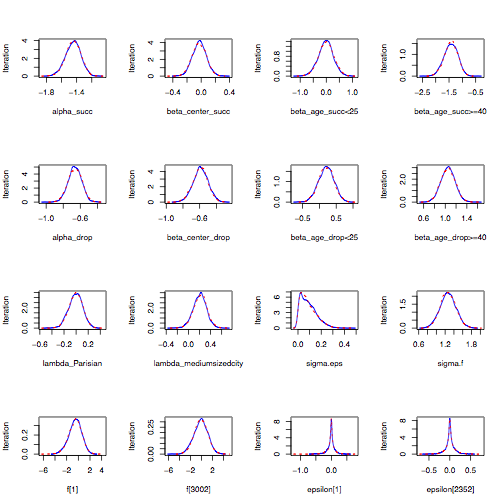


**Bayesian statistical hypothesis testing**

The following statistical hypothesis H_0_: λ_center_ =0 against H_1_: λ_center_ ≠0 was tested for both IVF centers according to Bayesian statistical theory methods. This led to comparison of the following two pairs of models using partial Bayes factors (BF) :

- M_0_: [λ_Paris_ = 0 and λ_Medium-sized city_ =0] against M_1_: [λ_Paris_≠0 and _λMedium-sized city_ =0] that allows testing H_0_: λ_Paris_ = 0 vs H_1_: λ_Paris_≠0 (when λ _Medium-sized city_ is fixed at 0)
- M_0_: [λ_Paris_ = 0 and λ_Medium-sized city_ =0] vs M_1_: [λ_Paris_=0 and λ_Medium-sized city_ ≠0] that allows testing H_0_: λ_Medium-sized city_ =0 vs H_1_: λ_Medium-sized city_ ≠0 (when λ_Paris_ is fixed at 0).

Interpretation of the value of the partial BF (at 2 log scale) for H_0_ against H_1_, denoted L_01_=2logBF_01_ .

- if L_01_belongs to [0, 2], no evidence against λ_center_ ≠0
- if L_01_ ≥2, positive evidence against λ_center_ ≠0 meaning that λ_center_ is non-significantly different from 0 (H_0_ is not rejected)
- if L_01_ ≥6, strong evidence against λ_center_ ≠0 meaning that λ_center_ is strongly non-different from 0 (H_0_ is not rejected)
- if L_01_ belongs to [-2,0], no evidence against λ_center_ =0
- if L_01_ ≤-2 , positive evidence against λ_center_ =0 meaning that λ_center_ is significantly different from 0 (H_0_ is rejected)
- if L_01_ ≤-6, strong evidence against λ_center_ =0 meaning that λ_center_ is strongly different from 0 (H_0_ is rejected)

**Analysis of Bayes factor sensitivity to priors**

Bayes factors are known to be sensitive to prior choices. A traditional approach to testing Bayes factor sensitivity to priors consists in computing Bayes factors from different prior distributions. Its major drawback is computational intensity as one MCMC algorithm has to be run for each tested prior. We therefore used an ingenious method for rapidly analyzing Bayes factor sensitivity to priors: we computed partial Bayes factors from more or less informative priors (and only one MCMC run) by performing a split test sample analysis as described elsewhere . We considered three ways to split the sample of 3002 women so as to define a learning sample (i.e., 2702, 1502 and 502 women) that would be more or less informative on the remaining test sample (i.e., 300, 1500, 2500 women). Our global results are insensitive to the three tested levels of prior information. Indeed, when comparing M_0_: [λ_Paris_ = 0 and λ_Medium-sized city_ =0] against M_1_: [λ_Paris_≠0 and λ_Medium-sized city_ =0], there is always positive evidence against λ_Paris_ ≠0 meaning that λ_Paris_ is non-significantly (NS) different from 0 (H_0_ is not rejected). When comparing M_0_: [λ_Paris_ = 0 and λ_Medium-sized city_ =0] against M_1_: [λ_Paris_=0 and λ_Medium-sized city_ ≠0], there is always positive evidence against λ_Medium-sized city_ =0 meaning that λ _Medium-sized city_ is significantly (S) different from 0 (H_0_ is rejected).

**Table A1**. Partial Bayes factors (at 2 log scale) for M_0_ against M_1_ computed from 100 test samples of 300 women. This corresponds to the specification of strongly informative priors (learning sample: 2702 women).

|  | M_1_: λ_Parisi_≠0  λ_Medium-sized city_ =0 | M_1_: λ_Paris_=0  λ_Medium-sized city_ ≠0 |
| --- | --- | --- |
| M_0_: λ_Paris_ = 0  λ_Medium-sized city_ =0 | 2.70 | -5.26 |

**Table A2**. Partial Bayes factors (at 2 log scale) for M_0_ against M_1_ computed from 100 test-samples of 1500 women. This corresponds to the specification of informative priors (learning sample: 1502 women).

|  | M_1_: λ_Paris_≠0  λ_Medium-sized city_ =0 | M_1_: λ_Paris_=0  λ_Medium-sized city_ ≠0 |
| --- | --- | --- |
| M_0_: λ_Paris_ = 0  λ_Medium-sized city_ =0 | 2.22 | -6.30 |

**Table A3**. Partial Bayes factors (at 2 log scale) for M_0_ against M_1_ computed from 100 test-samples of 2500 women. This corresponds to the specification of weakly informative priors (learning sample: 502 women).

|  | M_1_: λ_Paris_≠0  λ_Medium-sized city_ =0 | M_1_: λ_Paris_=0  λ_Medium-sized city_ ≠0 |
| --- | --- | --- |
| M_0_: λ_Paris_ = 0  λ_Medium-sized city_ =0 | 2.23 | -6.50 |

**Sensitivity analysis to prior choice**

We also conducted sensitivity analyses on the shared random effects models to assess the impact on inference of our choice of priors for the variance parameters **σ_ε_^2^** and **σ_f_^2^** and the regression coefficients. As an alternative set of hyperpriors for the variance parameters, we chose inverse gamma distributions IGamma(0.01,0.01), as commonly used due to their conjugacy property in the Bayesian framework. For the regression coefficients, we also considered the popular Zellner’s g-prior distributions , which are based on the inverse of empirical covariance matrix of the predictors. The parameters and random effects estimates were globally insensitive to these prior choices. This indicates that there is enough information in the data sources to learn about all the unknown quantities of our shared random effects model.

**Parameter estimates**

**Table A4.** Estimations of the baseline factors, the effects of woman’s age and IVF center (on the odds ratio scale), the variance parameters and the links between the success and treatment discontinuation processes for the monopoly and the non-monopoly center.

| Success process | | | Treatment discontinuation process | | |
| --- | --- | --- | --- | --- | --- |
| Parameter | Posterior mean  (Posterior standard deviation) | 95% credible interval | Parameter | Posterior mean  (Posterior standard deviation) | 95% credible interval |
| α^succ^ | 0.24 (0.02) | [0.19; 0.28] | α^drop^ | 0.52 (0.04) | [0.44; 0.60] |
| $\beta_{center}^{succ}$ | 0.98 (0.09) | [0.81; 1.18] | $\beta_{center}^{drop}$ | 0.56 (0.05) | [0.46; 0.66] |
| $\beta_{age}^{succ}$ < 25 | 1.03 (0.30) | [0.56; 1.73] | $\beta_{age}^{drop}$<25 | 1.26 (0.28) | [0.78; 1.91] |
| $\beta_{age}^{succ}$ 25 -29 | 1.16 (0.13) | [0.92; 1.44] | $\beta_{age}^{succ}$ 25-29 | 1.15 (0.10) | [0.96; 1.37] |
| $\beta_{age}^{succ}$ 35-39 | 0.56 (0.07) | [0.43; 0.70] | $\beta_{age}^{succ}$ 35-39 | 1.51 (0.13) | [1.28; 1.78] |
| $\beta_{age}^{succ}$ ≥ 40 | 0.25 (0.06) | [0.14; 0.38] | $\beta_{age}^{succ}$ ≥ 40 | 2.88 (0.38) | [2.21; 3.68] |
| **σ_f_** | 1.27 (0.18) |  | **σ_ε_** | 0.09 (0.07) | [0.004; 0.25] |
|  |  |  | **λ_Paris_** | 0.01 (0.11) | [-0.21; 0.23] |
|  |  |  | **λ_Medium-sized city_** | -0.21 (0.12) | [-0.45; -0.04] |

**Discussion of the model**

Convergence is slow for the variance parameters σ_f_^2^ and σ_ε_^2^ when fitting the current version of our shared random effects model with MCMC algorithms. Basically, the chains produced exhibit poor mixing due to a high level of autocorrelation for such variance parameters. Consequently, very large samples of values had to be simulated to obtain accurate Bayesian estimations meaning high computation times. This drawback has already been reported by Browne et al. in the specific case of multilevel discrete time models. Hence, our shared random-effects model can also be seen as two multilevel discrete time survival models running together. Basically, we have just expanded the data set - meaning that each time to event was converted to a sequence of discrete responses, usually binary, indicating for each attempt whether an event (live-birth or treatment discontinuation) has occurred - so that the models can be simply cast as standard mixed logistic regression models. Such a technical difficulty may also be due to the fact that shared random effects models are constrained latent variable models, in addition to the well-known problem of the Bayesian estimation of small variance parameters (e.g., here σ_ε_^2^) with MCMC algorithms . Note that there are some methods to increase the computational efficiency of MCMC estimation of our shared random effects model, such as the parameter expansion approach proposed by Kinney and Dunson .

**References**

1. Robert CP, Casella G: *Monte Carlo Statistical Methods*. 2nd edition: Springer-Verlag; 2004.

2. Lunn DJ, Thomas A, Best N, Spiegelhalter D: **WinBUGS - A Bayesian modelling framework: Concepts, structure, and extensibility**. *Stat Comput* 2000, **10**(4):325-337.

3. Gelman A: **Prior distributions for variance parameters in hierarchical models.** *Bayesian Analysis* 2006, **1**(3):515-533.

4. Brooks SP, Gelman A: **Alternative methods for monitoring convergence of iterative simulations**. *J Comput Graph Stat* 1998, **7**:434-455.

5. Kass RE, Raftery AE: **Bayes Factors**. *J Am Stat Assoc* 1995, **90**(430):773-795.

6. Ancelet S, Etienne M-P, Benoît H, Parent E: **Modelling spatial zero-inflated continuous data with an exponentially compound Poisson process** *Environ Ecol Stat* 2010, **17**:347-376.

7. Zellner A: **On assessing prior distributions and Bayesian regression analysis with g-prior distributions.** In: Goel PK, Zellner A (eds), Bayesian Inference and Decision Techniques: Essays in Honor of Bruno de Finetti. North-Holland Elsevier edition. Amsterdam; 1986.

8. Browne WJ, Steele F, Golalizadeh M, Green MJ: **The use of simple reparameterizations to improve the efficiency of Markov chain Monte Carlo estimation for multilevel models with applications to discrete time survival models**. *J R Stat Soc Ser A Stat Soc* 2009, **172**(3):579-598.

9. Gelman A: **Parametrization and Bayesian modeling.** *J Am Stat Assoc* 2004, **99**:537-545.

10. Kinney SK, Dunson DB: **Fixed and random effects selection in linear and logistic models**. *Biometrics* 2007, **63**(3):690-698.
